# Two Cases of Thoracentesis

**Published:** 1857-06

**Authors:** 


*Two Cases of Thoracentesis, performed for Acute and very considerable Pleuritic Effusion and followed by Recovery.* (L'Union Medicale, tome x., Nos. 147, 148.)

The cases occurred at the Hôpital St. Antoine, under the care of M. Aran. The patients were men respectively of the ages of twenty-six and thirty-nine years. Ia the first, the pleurisy affected the left side, and the effusion was so considerable as to force the heart above an inch (three centim.) beyond the right margin of the sternum; in the second, the right side was affected, and the heart was pushed over to the left, so that the heart-dulness only commenced at the left edge of the sternum, The dislocation of the heart forms one of the chief sources of the danger accompanying pleuritic effusion, and may therefore be regarded as an argument in favor of paracentesis. Paracentesis was accomplished in the former case a few days after the patient's admission to the hospital, when he had been about four weeks ill. One thousand two hundred grammes (above twenty-six ounces) were evacuated; the immediate relief was great, and an entire recovery followed, so that he was discharged, cured, three weeks after. In the second case, the operation was performed four weeks after the commencement of the illness, and a week after the patient's admission. The amount of fluid evacuated was 2560 grammes (about fifty-five ounces). A fortnight after, the patient is reported to be doing perfectly well, being retained in the hospital simply as a matter of precaution.

In neither of the cases was there much fever on the day on which the puncture was made; the pulse was eighty-four in the first, sixty in the second patient; the former presented twenty-four, the latter twenty, respirations in the minute. They had some appetite, and probably neither patient considered himself dangerously ill; still, the extent of the effusion left no doubt that their malady was a very serious one. This recovery was the most rapid and complete, as regards the expansion of the compressed lung, in the second case—still, in both, the lung that had been affected, was restored nearly to the normal condition. The first at his discharge is reported to have retained only a slight dulness, with a somewhat feeble respiratory murmur on the left side; while the second, eight days after the operation, presented nothing but a slight diminution of the respiratory murmur at a circumscribed spot at the lower and outer part of the affected side.

